# Mitigation of Salinity Stress in Wheat Seedlings Due to the Application of Phytohormone-Rich Culture Filtrate Extract of Methylotrophic Actinobacterium ***Nocardioides*** sp. NIMMe6

**DOI:** 10.3389/fmicb.2020.02091

**Published:** 2020-09-18

**Authors:** Kamlesh K. Meena, Utkarsh M. Bitla, Ajay M. Sorty, Dhananjaya P. Singh, Vijai K. Gupta, G. C. Wakchaure, Satish Kumar

**Affiliations:** ^1^ICAR-National Institute of Abiotic Stress Management, Baramati, India; ^2^ICAR-National Bureau of Agriculturally Important Microorganisms, Mau, India; ^3^Department of Chemistry and Biotechnology, Tallinn University of Technology, Tallinn, Estonia

**Keywords:** *Nocardioides* sp., IAA, salicylic acid, seed-priming, wheat, salinity

## Abstract

Salinity stress is an important plant growth limiting factor influencing crop productivity negatively. Microbial interventions for salinity stress mitigation have invited significant attention due to the promising impacts of interactive associations on the intrinsic mechanisms of plants. We report the impact of microbial inoculation of a halotolerant methylotrophic actinobacterium (*Nocardioides* sp. NIMMe6; LC140963) and seed coating of its phytohormone-rich bacterial culture filtrate extract (BCFE) on wheat seedlings grown under saline conditions. Different plant-growth-promoting (PGP) attributes of the bacterium in terms of its growth in N-limiting media and siderophore and phytohormone [indole-3-acetic acid (IAA) and salicylic acid] production influenced plant growth positively. Microbial inoculation and priming with BCFE resulted in improved germination (92% in primed seeds at 10 dS m^–1^), growth, and biochemical accumulation (total protein 42.01 and 28.75 mg g^–1^ in shoot and root tissues at 10 dS m^–1^ in BCFE-primed seeds) and enhanced the activity level of antioxidant enzymes (superoxide dismutase, catalase, peroxidase, and ascorbate peroxidase) to confer stress mitigation. Biopriming with BCFE proved impactful. The BCFE application has further influenced the overexpression of defense-related genes in the seedlings grown under salinity stress condition. Liquid chromatography–mass spectrometry-based characterization of the biomolecules in the BCFE revealed quantification of salicylate and indole-3-acetate (Rt 4.978 min, *m/z* 138.1 and 6.177 min, 129.1), respectively. The high tolerance limit of the bacterium to 10% NaCl in the culture media suggested its possible survival and growth under high soil salinity condition as microbial inoculant. The production of a high quantity of IAA (45.6 μg ml^–1^ of culture filtrate) by the bacterium reflected its capability to not only support plant growth under salinity condition but also mitigate

stress due to the impact of phytohormone as defense mitigators. The study suggested that although microbial inoculation offers stress mitigation in plants, the phytohormone-rich BCFE from *Nocardioides* sp. NIMMe6 has potential implications for defense against salinity stress in wheat.

## Graphical Abstract

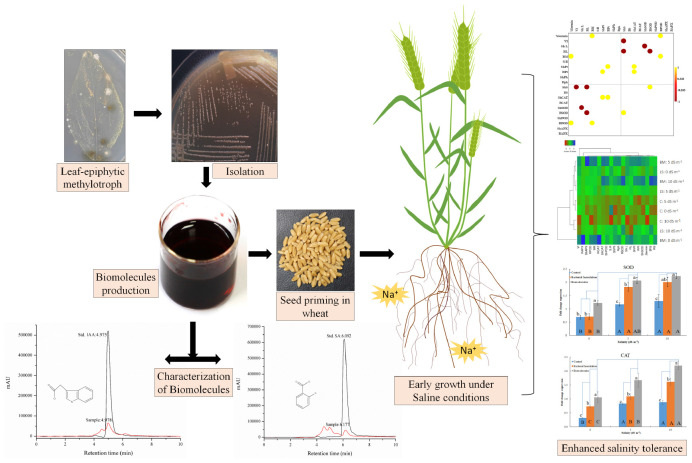

## Introduction

Salinity, among the most agonizing abiotic stresses, is responsible for significantly declining agricultural productivity (Allakhverdiev et al., 2000). Saline conditions develop predominantly due to natural processes or frequent irrigation with saline water (Mishra et al., 2016). The growth stages of crop plants right from seed germination and plant development up to seed production are highly susceptible to saline conditions (Kumar et al., 2015; Sorty et al., 2016). Exposure of plants to salinity during the early developmental stages negatively influences the overall process of water and nutrient uptake and thus worsen crop growth and productivity (James et al., 2002; Dodd and Pérez-Alfocea, 2012). High salt concentration limits root development by impairing physiological and metabolic balance and significantly reducing the rate of seed germination (Munns et al., 2006). Excessive accumulation of sodium ions in surviving plants interfere with the photosynthetic mechanism in the leaves and generate oxidative stress due to the accumulation of reactive oxygen species (ROS) (Acosta-Motos et al., 2017). A high concentration of ROS damages cellular compartmentalization by causing peroxidation of membrane lipids and leads to the development of early leaf senescence forcefully (James et al., 2002).

Plants employ a range of intrinsic mechanisms at the cellular level to combat stress conditions (Meena et al., 2017). Under associative interaction with the plants, microbial species play an important role in inducing stress-responsive mechanisms which go in parallel to the plant’s own responses (Kumar and Verma, 2018). Microbial associations directly influence the expression of several stress-responsive genes, activation of antioxidant enzymes, and accumulation of proteins, compatible solutes, and bioactive compounds that cumulatively add to enhance the stress tolerance capability of plants (Kaushal and Wani, 2016). Currently available strategy to harness the efficiency of microbial inoculation for crop plants grown under abiotic stress condition is mainly based on inoculating either individual microbial species or their consortia as live inoculants (Mazzoli et al., 2017; Sorty et al., 2018; Bakka and Challabathula, 2020). It has been established that the exogenous application of metabolites and phytohormones enhances plant-inherent defense systems that subsequently help plants in stress mitigation (An et al., 2014; Sharma et al., 2019). The biomolecules may act as signaling molecules that regulate cross-talk during plant–environment interactions (Bitla et al., 2017; Ku et al., 2018; Ahmad et al., 2019). The phenomenon of the subsequent protection against stresses due to the application of organic, inorganic, or biostimulants is known as molecular priming (Kerchev et al., 2020). If explored, the potentiality of biomolecules of microbial origin can find a significant role in stress mitigation and growth promotion strategy in plants. Such surge of interest could offer strategic dimensions for wide-scale implications of microbial biomolecules in crops grown in saline areas or exposed to drought (Bulgari et al., 2019).

Seed priming is an important approach that is being widely used in raising crop plants for improving germination, seedling vigor, growth, development, yield, and stress tolerance (Zheng et al., 2016; Hussain et al., 2018). Priming of seeds with phytohormones (hormopriming), osmotic agents (osmopriming), and hydration (hydropriming) has remained beneficial in crop production strategy under drought and saline conditions (Paparella et al., 2015; Sorty et al., 2016). There remains a dearth of knowledge on the potential of microbial products as seed priming agents for mitigating abiotic stress in crop plants (O’Callaghan, 2016; Egamberdieva et al., 2017). We have isolated methylotrophic halotolerant actinobacterium *Nocardioides* sp. NIMMe6 from soybean leaf surface. The bacterium possesses plant-growth-promoting (PGP) and phytohormone-producing capabilities. It has shown its survival at 10% NaCl concentration in culture media and subsequently produced culture filtrate rich in phytohormones, thereby reflecting that it may be employed as an inoculant in saline conditions. Wheat, cultivated in many parts of India, is highly vulnerable to saline conditions (Hampson and Simpson, 1990). We have shown that the application of the bacterium on wheat as an inoculant and through seed priming of the phytohormone-rich bacterial culture filtrate extract (BCFE) resulted in the activation of plant-intrinsic responses against salinity stress. This study describes specific biochemical and molecular mechanisms involved in offering mitigation of salinity stress in wheat seedlings after priming with BCFE and microbial inoculation.

## Materials and Methods

### Identification and Functional Characterization of the Bacterium

Fresh leaves of soybean crop grown at an experimental farm of the ICAR-National Institute of Abiotic Stress Management campus (Baramati, India; N18.1388, E74.5063) were collected for the isolation of the bacterium. The impressions of the dorsal and the ventral sides of healthy, juvenile soybean leaves were taken on the ammonium mineral salt agar medium (Omer et al., 2004) amended with 5.0% NaCl (Knief et al., 2008). After incubation at 30 ± 2°C for 72 h, colonies were purified and pure stock was maintained in 50% (v/v) glycerol at −80°C.

The genomic DNA was isolated using Ultraclean Microbial DNA isolation kit (MoBio, Carlsbad, United States). Polymerase chain reaction (PCR) amplification of 16S rRNA gene was carried out (Sorty et al., 2016), and the PCR product was purified using QIAquick gel extraction kit (Qiagen), followed by sequencing in ABI 3130*xl* automated genetic analyzer (Applied Biosystems, United Kingdom). Sequence analysis was performed using MEGA7 (Kumar et al., 2016). BLAST was performed using National Center for Biotechnology Information – Basic Local Alignment Search Tool^1^. Sequence alignment with the datasets of related organisms was done with the help of ClustalW. A phylogenetic tree was constructed using neighbor joining method (Tamura et al., 2004). The sequence was finally submitted to DNA Data Bank of Japan.

Biolog-based substrate utilization assay was used for the metabolic characterization of the isolate using Biolog GEN III (Biolog Inc., United States) protocol. The bacterial culture (100 μl) was inoculated in a 96-well Biolog GEN III plate having a specific carbon source per well and incubated at 30 ± 2°C. Growth and substrate metabolism was recorded at 12-h intervals using Biolog MicroStation. The culture was grown with different carbon sources including amino acids, carboxylic acids, sugars, and organic acids. Metabolic intensity was tracked colorimetrically using tetrazolium redox dye, which developed a purple color.

### Bacterial Growth on N-Deficient Media

The ability of the isolate to grow in nitrogen-deficient media was detected using Ashby’s N-free mannitol agar (Sen and Sen, 1965). A 10-μl aliquot of freshly grown culture was inoculated on the agar media plates and incubated at 30 ± 2°C for 48 h. Development of visible growth on the plates was noticed.

### Phosphate Solubilization

Phosphate solubilization by the isolate was detected on Pikovskaya’s agar (Nautiyal, 1999). A freshly grown culture was spot-inoculated (10 μl) on three separate plates, and the inverted plates were incubated at 30 ± 2°C for 48 h. P solubilization was monitored in terms of the development of a clear halo zone surrounding the microbial growth.

### Siderophore Production

Production of siderophore was detected using chrome azurol sulfonate (CAS) agar (Schwyn and Neilands, 1987). Briefly, 10 μl of log culture of the bacterium was spot-inoculated on freshly prepared CAS agar plates and incubated at 30 ± 2°C for 48 h to observe qualitative siderophore production.

### Bacterial Salt Tolerance and Growth Curve Analysis

The salt tolerance ability of the strain was detected using stepped gradient concentration of NaCl from 0 to 30% in lysogeny agar medium (Sorty et al., 2016). Freshly grown bacterial culture (10 μl) was spot-inoculated on agar plates amended with NaCl concentration ranging from 1 to 30% and incubated at 30 ± 2°C for 7 days. Appearance of visible growth on the agar medium was recorded. The maximum NaCl concentration showing a visible growth of the isolate was selected as upper end for growth curve analysis.

Growth curve was analyzed in lysogeny broth (LB) medium amended with 1% gradual salt (NaCl) concentration. Freshly grown bacterial cells (100 μl) were inoculated in 100 ml of the medium and incubated at 30 ± 2°C at 150 rpm in a shaker incubator. Three flasks were maintained for each salt concentration. The growth of the bacterium was monitored in terms of change in OD_600__nm_ and plotted against time to obtain NaCl-dependent growth curve.

### Exopolysaccharide Production

The exopolysaccharide (EPS) production ability of the bacterium was detected on tryptic soy agar (TSA). Freshly grown bacterial culture (10 μl) was inoculated on three separate TSA plates and incubated at 30 ± 2°C for 48 h for qualitative estimation of EPS (Vardharajula et al., 2011).

### Indole Acetic Acid Production

The bacterium was grown at 30°C in LB medium supplemented with 120 mM methanol for 48 h at 150 rpm. Spectrophotometric quantification of indole-3-acetic acid (IAA) was done by using Salkowski reagent (Ehmann, 1977). In brief, 0.5 ml of culture filtrate, obtained by pelleting the cells at 7,000 rpm for 5 min, was mixed with 2 ml of Salkowski reagent (2% of 0.5 M FeCl_3_ in 35% HClO_4_). To this mixture, 200 μl of orthophosphoric acid was added with mixing, and the tubes were kept in the dark for 30 min at room temperature. Absorbance was recorded at 530 nm, and calibration was obtained against pure IAA (Fisher Scientific, United States).

### Preparation of Bacterial Culture Filtrate Extract

The bacterium was grown in LB, a nutritionally rich culture medium most commonly used for growing bacteria for molecular biological (Miller, 1972) and secondary metabolite including phytohormone production studies (Cox et al., 2018). After attaining culture growth at 30 ± 2°C for 36 h at 150 rpm, bacterial cells were separated by centrifugation at 7,830 rpm (Eppendorf 5430R) to obtain a cell-free culture filtrate. The culture filtrate was then mixed with preconditioned xad-16 resin (10% w/v) (Sigma Aldrich, United States) for increasing titer of the extraction of metabolites due to its high adsorption capacity (González-Menéndez et al., 2019). A slurry of the culture filtrate and the xad-16 resin was prepared at 150 rpm for 2 h at room temperature and then poured into a chromatographic glass column (10 mm diameter × 300 mm length) having a built-in sintered disk at the bottom. The column was washed thrice with ethyl acetate and methanol (1:1 v/v). The solvent was evaporated at room temperature, and the BCFE was stored at 4°C for further analysis. A separate set of slurry using xad-16 resin was also prepared with the same LB medium that was used for growing bacterial cells and was subjected to column separation exactly as detailed above. The medium extract thus obtained served as reference blank to ensure that the identified metabolite constituents are produced and excreted into the culture filtrate by the bacterial cells only.

### Analysis of Phytohormones Using HPLC and LC–MS

Quantitative estimation of phytohormone in the BCFE was performed using high-performance liquid chromatography (HPLC). An aliquot (10 μl) of BCFE dissolved in methanol was injected onto the RP C-18 column (4.6 mm i.d. × 250 mm length; particle size, 3 μm) (Spincotech, India) using the auto-sampler (SIL 30AC) fitted with the HPLC system (Nexera X2; Shimadzu Corp., Japan) running with the mobile phase [methanol (A); 0.1% formic acid in water (B); final composition A:B, 80:20 v/v]. The system was equipped with a photodiode array detector (SPD M20A) and a column oven (CTO 20AC) maintained at 30°C throughout the analysis. The mixture was eluted under isocratic mode at 0.7 ml min^–1^. Compounds were characterized on the basis of their retention time (Rt) and co-elution with the standard compounds. The medium extract was also analyzed under a similar set of conditions to exclude traces of IAA or salicylic acid, if any, coming from the medium constituents.

The identity of the compounds was authenticated with the help of mass spectrometry (MS) (Agilent 1200 quadrupole LC–MS system), in which the sample was injected in absolute methanol at a flow rate of 0.5 ml min^–1^. The compounds were identified on the basis of their *m/z* values.

### Gnotobiotic Experiment Under Saline Conditions

The impact of microbial inoculation and BCFE priming was investigated on wheat grown in gnotobiotic condition under a saline environment. Sterile petri dishes having different levels of salinity [electrical conductivity (EC) 0, 5, and 10 dS m^–1^, maintained using NaCl; pH 7.0 ± 0.2] were prepared with agar-water (0.7%). Wheat seeds (var. Netravati NIAW1415 – recommended for rainfed and restricted irrigation regimes having normal soil conditions) were surface-sterilized using 5% (v/v) sodium hypochlorite solution (Sauer and Burroughs, 1986). Residual hypochlorite was removed by washing three times with sterile Milli Q water. Bacterial cell suspension (∼10^9^ cfu ml^–1^) in 1% aqueous carboxymethyl cellulose (CMC) as binding agent was given for microbial inoculation on the wheat seeds. Seed priming of wheat was performed by soaking the seeds with a solution of BCFE prepared in 1% CMC in water (at 1 mg extract per gram of seeds). The seeds were dried in laminar hood on a sterile polythene sheet, sown on the Petri dishes in four replicates (*n* = 4), and incubated in the dark for 72 h at 20°C followed by 12-h dark and light cycle until 7 days. Uninoculated or non-primed seeds served as control.

Germination percentage, length of shoot and root, seedling vigor index, total biomass, and shoot–root ratio were determined from freshly harvested seedlings.

### Biochemical Analysis of Wheat Seedlings

#### Total Protein Content

Total protein content in shoot and root tissue was measured according to Bradford (1976). The tissue extracts prepared for enzyme estimation were mixed with Bradford reagent and allowed to react in the dark for 15 min, and the absorbance was recorded at 595 nm. Total content of protein was quantified using bovine serum albumin as standard.

#### Estimation of Total Sugar

The total sugar content of shoot and root tissue was estimated using anthrone reagent (Yemm and Willis, 1954). A crushed sample (50 mg) was treated with 2.5 M HCl in boiling water bath for 3 h. The acid was neutralized with an excess of sodium carbonate, and the total content was diluted to 50 ml. The clear supernatant (1 ml) was mixed with 4 ml of freshly prepared, ice-cold anthrone reagent (0.2% anthrone in 95% H_2_SO_4_). After thorough mixing, the content was kept in boiling water bath for 10 min and cooled, and the absorbance was recorded at 630 nm. Total sugar content was calculated as the equivalents of glucose.

#### Total Polyphenolic Content

Total polyphenolic content (TPC) was measured according to Singleton et al. (1999). For this, 100 mg of tissue sample was macerated in 2 ml of chilled 80% methanol. The debris was removed by centrifugation at 14,000 rpm for 15 min, and 100 μl supernatant was mixed with Folin Ciocalteu reagent (1 N). The reaction mixture was kept in boiling water bath for 1 min and cooled. The absorbance was noted at 650 nm by taking catechol as the standard.

#### Estimation of Antioxidant Enzymes

Enzyme extract of shoot and root was prepared by crushing 1 g of tissue in ice-cold phosphate buffer [4.0 ml of 100 mM, 0.5 mM ethylenediaminetetraacetic acid (EDTA), pH 7.5]. Tissue debris was removed by centrifugation at 14,000 rpm, and the supernatant was stored at −20°C.

Catalase activity was determined with the reaction mixture (3 ml) containing potassium phosphate buffer (50 mM, pH 7.0), hydrogen peroxide (12.5 mM), and the enzyme extract (50 μl) (Luck, 1974). The enzyme reaction was monitored in terms of decreasing absorbance at 240 nm for 1 min.

Superoxide dismutase activity was determined in 3-ml reaction mixture containing methionine (13.33 mM), EDTA (0.1 mM), phosphate buffer (50 mM), sodium carbonate (50 mM), nitro blue tetrazolium chloride (75 μM), 100 μl of enzyme, and riboflavin (2.0 μM) (Dhindsa et al., 1981). The mixture was illuminated for 15 min under white fluorescent light, followed by dark exposure for 15 min. The absorbance was recorded at 560 nm. Illuminated and non-illuminated reaction mixtures without enzyme served as control. Fifty percent reduction of absorbance compared to control was considered as one unit of the enzyme.

Peroxidase activity was determined in 3 ml of the reaction mixture containing potassium phosphate buffer (50 mM, pH 6.1), guaiacol (16 mM), hydrogen peroxide (2.0 mM), and enzyme extract (100 μl) (Reddy et al., 1995). Progress of the reaction was monitored in terms of the formation of guaiacol tetramers at 470 nm.

Ascorbate peroxidase activity was estimated as described by Nakano and Asada (1981). To the 3.0 ml of reaction mixture having potassium phosphate buffer (50 mM, pH 7.0), ascorbic acid (0.5 mM), EDTA (0.1 mM), and hydrogen peroxide (0.1 mM), the enzyme extract (100 μl) was added for determining ascorbate peroxidase (APX). The change in absorbance of the reaction mixture for 30 s was recorded at 290 nm.

#### Quantitative Real-Time PCR

Fresh leaf tissues were frozen in liquid nitrogen and subjected to RNA extraction using RNeasy Plant Mini Kit (Qiagen, Leusden, Netherlands) as per the manufacturer’s instructions. Purified RNA was used for the synthesis of cDNA using Verso cDNA Synthesis Kit (Thermo Scientific, United States). Amplification and quantitation of reference and target genes were done in a 96-well (cfx96) real-time PCR system (Bio-Rad, United States) using DyNAmo ColorFlash SYBR Green qPCR master mix (Thermo Scientific, United States). The reaction program consisted of initial denaturation at 95°C for 10 min, followed by 40 cycles of denaturation at 95°C for 15 s, annealing/extension at 60°C for 60 s with the lid temperature 105°C, and fluorescence recording at each cycle. The primers used for the quantification of gene transcripts related to the antioxidant enzymes were catalase (CAT; forward 5′-CCATGAGATCAAGGCCATCT-3′, reverse 5′-ATCTTACATGCTCGGCTTGG-3′), manganese superoxide dismutase (MnSOD; forward 5′-CAGAG GGTGCTGCTTTACAA-3′, reverse 5′-GGTCACAAGAGGG TCCTGAT-3′), APX (forward 5′-GCAGCTGCTGAAGGA GAAGT-3′, reverse 5′-CACTGGGGCCACTCACTAAT-3′) (Baek and Skinner, 2003), and peroxidase (POD; 5′-CAG CGACCTGCCAGGCTTTA-3′, reverse 5′-GTTGGCCC GGAGAGATGTGG-3′) (Jiang et al., 2012). The Ct value of a constitutive reference transcript of wheat actin (5′-CGAAACCTTCAGTTGCCCAGCAAT-3′, reverse 5′-ACCATCACCAGAGTCGAGCACAAT-3′) (Dong et al., 2013) was used to normalize gene expression.

### Statistical Analysis

Unless described separately, all the experiments were conducted in triplicate. The numerical data were statistically analyzed using two-way analysis of variance with *post hoc* Duncan’s multiple-range test using SPSS 16.0 (Windows 8.0). A clustered heat map was plotted to establish the relationship among the measured physicochemical attributes of the wheat seedling. Pearson’s correlation analysis of the treatments was also performed using SPSS 16.0 and Past3. Difference at 95% confidence level was considered as significant.

## Results

### Identification and Functional Characterization of the Bacterium

**FIGURE 1:**
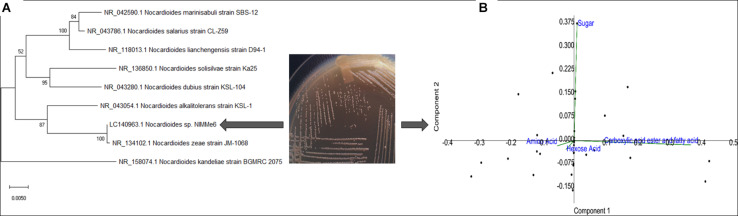
Characterization of the methylotrophic bacterium from soybean leaf surface. Phylogenic positioning of the strain using MEGA 7 (neighbor-joining, bootstrap: 1,000 replicates) revealed its identity as *Nocardioides* sp. **(A)**. Biolog GEN III profiles of the strain indicated high metabolic plasticity **(B)**.

The bacterium isolated from the leaf surface of the soybean crop was identified as *Nocardioides* sp. NIMMe6 (accession: LC140963) on the basis of 16S rRNA gene sequence similarity (Figure 1A and Table 1). BIOLOG Gen III assay revealed the metabolic flexibility of *Nocardioides* sp. NIMMe6. The bacterium utilized amino acid and hexose sugar as carbon source with high affinity. It also metabolized carboxylic acid and derivatives, carboxylic esters, and fatty acids (Figure 1B and Supplementary Figure S3). The identified bacterium was able to grow on Ashby’s N-free mannitol agar media, thereby qualitatively showing its ability to manage nitrogen from the atmosphere and sustain under N-limited conditions. It has failed to solubilize phosphate but has shown an orange halo zone in the CAS medium, indicating siderophore production ability (Table 1). The bacterium was tolerant to 10% NaCl concentration in the medium, with optimal growth at 4%, and was negative to epoxypolysaccharide production (Table 1). The growth of the bacterium in the salt (NaCl) concentration ranging from 1 to 10% exhibited a characteristic trend, with reduced growth and an extended lag-time at no salt as well as at high salt (8–10%). However, the bacterium showed an increasing trend of growth from 2% salt and continued until 4% of salt, where peak growth was noted, after which the growth started decreasing and continued until 10% of NaCl (Supplementary Figure S2).

**TABLE 1:** Identification of the bacterial strain and its functional attributes relating to plant growth promotion.

Strain	Identity	GenBank accession	N_2_ fixation	PO_4_ solubilization	Indole-3-acetic acid (μg/ml)	EPS	Siderophore	NaCl tolerance
M6	*Nocardioides* sp. *NIMMe6*	LC140963	++	−	45.6	−	+	0–10%

### Quantification of IAA and SA in BCFE

**FIGURE 2:**
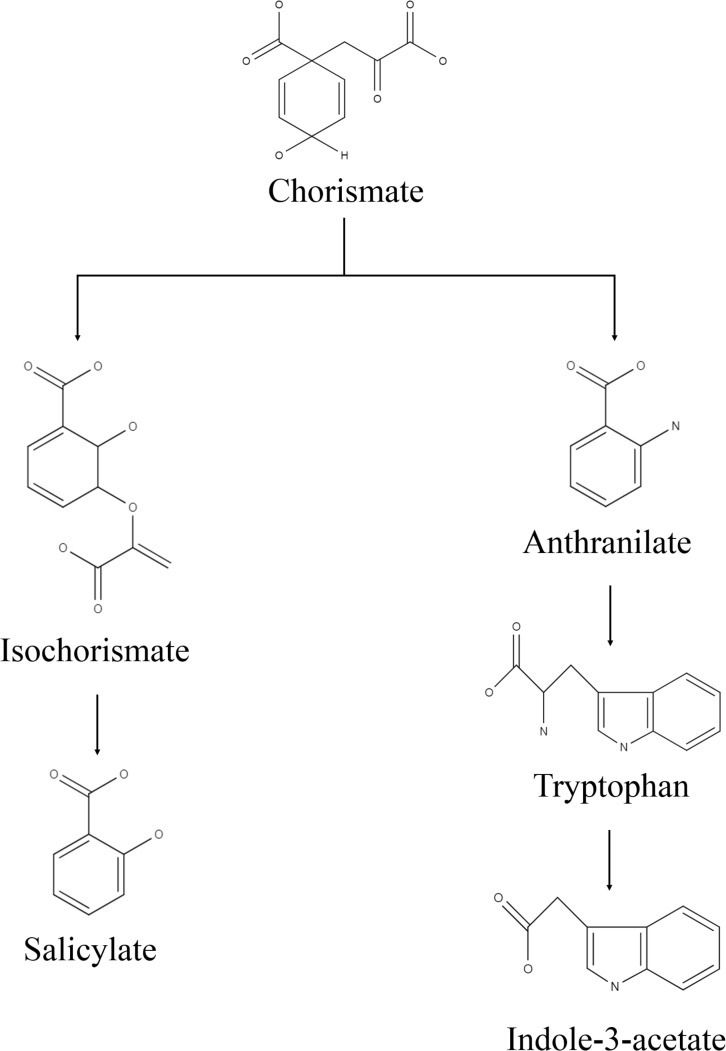
Predicted biosynthesis pathway implemented by the isolate for production of indole-3-acetic acid and salicylic acid.

Bacterial culture filtrate extract was obtained in the solution of ethyl acetate and methanol (1:1 v/v), which was passed through the BCFE-trapped XAD 16 resin. XAD resin has an excellent capacity to enrich different biological molecules including phenolic compounds, flavonoids, peptides, antimicrobial, pharmaceuticals, etc., and has been extensively used for the selective extraction of biomolecules from complex microbial culture media (Liu et al., 2016; Salazar et al., 2017; González-Menéndez et al., 2019) and other biological mixtures, such as adlay bran (Yang et al., 2016), apple skin Wang et al. (2020), *Glycyrrhiza* leaves (Dong et al., 2015), and wheat gluten hydrolysates (Yang et al., 2018), as well as extraction of pharmaceuticals in wastewater (Yu and Wu, 2011). Thus, to achieve maximal entrapment of microbial secretions and minimize the interference of residual medium impurities in the crude biomolecule mixture, we specifically used XAD-16 resin to trap the biomolecules secreted by *Nocardioides* sp. in LB medium. Due to the presence of tryptone and yeast extract, the LB medium allowed a luxurious production of biomolecules by providing multiple kinds of substrates, particularly in the form of amino acid precursors. The recovery of the dried BCFE was 16.8 mg l^–1^. *Nocardioides* sp. NIMMe6 produced and secreted into the medium a high amount of IAA (45.6 μg ml^–1^ of culture filtrate) (Supplementary Figures S1A,B). The secretion of IAA into the culture medium by *Nocardioides* sp. NIMMe6 was authenticated by HPLC (Rt 4.978 min) and MS spectra (*m/z* 129.1) (Supplementary Figure S1A). The bacterium also secreted salicylic acid (9.37 μg ml^–1^) into the culture medium, which was further verified by HPLC (Rt 6.177 min) and MS analysis (*m/z* 138.1) (Supplementary Figure S1B). Both of these prominent secondary metabolites, of which IAA is a phytohormone and salicylic acid is a signaling molecule, have been considered as elicitors in plants against abiotic stresses (Sorty et al., 2016; Ding and Ding, 2020; Figure 2).

### Impact of Bacterium Inoculation and BCFE Priming on Wheat

Inoculation with *Nocardioides* sp. NIMMe6 and seed priming with the BCFE resulted in a substantial impact on wheat grown under salinity stress condition (Table 2). BCFE priming enhanced seed germination (94.0% at 5 dS m^–1^) equivalent to that of non-saline control. High salinity condition of 10 dS m^–1^ lowered germination to 92% instead in BCFE-primed seeds. The bacteria-inoculated seeds also showed a germination percentage in the range of 91–92%, with no prominent diminishing effect of saline conditions. However, non-inoculated and non-primed seeds showed a significant reduction in germination at 10 dS m^–1^ compared to non-saline condition (Table 2). Vigor index (VI), which reflects the ability of seeds to produce normal seedlings, was improved due to seed priming with BCFE than in inoculated and non-inoculated conditions. Compared to the primed seeds (VI 1,820.9 at 5 dS m^–1^), the bacteria-inoculated seeds showed a VI of 1,514.5, while non-inoculated seeds resulted in a VI of 1,241.7. It indicated a better performance of BCFE seed priming than microbial inoculation (Table 2). A similar trend was also evident in the case of shoot and root length, shoot–root ratio, and biomass accumulation. On these parameters, the performance of BCFE seed priming under saline and non-saline condition was high compared to bacterial inoculation and non-inoculation (Table 2).

**TABLE 2:** Growth attributes of salinity-stressed wheat seedlings under the influence of the treatments with *Nocardioides* sp. and bacterial culture filtrate extract (BCFE).

EC (dS m^–1^)	Treatment	Germinationa^a^,%	Vigor index^b^	Shoot length^c^	Root length^d^	Biomass^d^	Shoot/root^e^
**0**	*Nocardioides* sp.	92.50 ± 1.73^ab^	1,700 ± 127.40*ab*	10.03 ± 0.39^ab^	8.34 ± 0.98^b^	0.014 ± 0.003 a-c	1.211 ± 0.102^ab^
5		92.00 ± 3.27^ab^	1,514.5 ± 169.29*bc*	8.24 ± 1.45^c^	8.20 ± 0.16^b^	0.015 ± 0.004^ab^	1.006 ± 0.176^c^
10		91.75 ± 2.50^ab^	1,018.4 ± 195.60e	5.84 ± 1.36^d^	5.25 ± 0.71^d^	0.012 ± 0.001^bc^	1.104 ± 0.114^bc^
0	BCFE	94.00 ± 5.16^a^	1,875 ± 138.08a	10.37 ± 0.52^a^	9.58 ± 0.66^a^	0.017 ± 0.002^a^	1.084 ± 0.041^bc^
5		94.00 ± 6.93^a^	1,820.9 ± 167.89a	9.93 ± 0.47^ab^	9.43 ± 0.32^a^	0.017 ± 0.004^a^	1.052 ± 0.029^bc^
10		92.00 ± 7.30^ab^	1,558 ± 156.94*bc*	8.98 ± 1.11^bc^	7.96 ± 0.28^b^	0.014 ± 0.001 a-c	1.132 ± 0.165^bc^
0	Control	91.00 ± 3.83^ab^	1,373 ± 109.50*cd*	8.56 ± 0.41^c^	6.51 ± 0.62^c^	0.011 ± 0.001^bc^	1.325 ± 0.150^a^
5		85.50 ± 7.55^bc^	1,241.7 ± 179.55d	6.58 ± 0.52^d^	7.90 ± 0.53^b^	0.012 ± 0.001^c^	0.832 ± 0.040^d^
10		81.25 ± 1.89^c^	764.5 ± 53.35^f^	3.27 ± 0.31^e^	6.14 ± 0.37^c^	0.011 ± 0.002^c^	0.534 ± 0.058^d^
	Mean square error	24.796	22457.008	0.706	0.322	5.04E-006	0.012
	*R*^2^	0.465	0.879	0.902	0.888	0.610	0.835

*Values in the same data series represented by different letters are significantly different (*p* = 0.05) in Duncan’s multiple-range test. ^*a*^Percent of seeds successfully germinated. ^*b*^Seedling length × germination percentage. ^*c*^Length in millimeters. ^*d*^Weight in milligrams (dry). ^*e*^Ratio obtained by dividing the shoot length with the root length.*

### Biochemical Analysis of Wheat Seedlings

Priming of seeds with BCFE not only enhanced seed quality and plant growth parameters but also increased the content of protein, sugars, and total phenolics in shoot and root as compared to bacteria-inoculated and non-inoculated plants under salinity stress (Table 3). It was apparent that plants grown with bacterial inoculation and BCFE priming under saline conditions (5 and 10 dS m^–1^) accumulated a high content of protein, sugar, and total polyphenolics in comparison to non-saline, non-inoculated, and non-primed plants.

**TABLE 3:** Biochemical characteristics of wheat seedlings treated with *Nocardioides* sp. and its bacterial culture filtrate extract (BCFE) under various salinity stress conditions.

		Protein^a^		Phenolic compounds^a^		Sugar^a^	
EC (dS m^–1^)	Treatment	Shoot	Root	Shoot	Root	Shoot	Root
0	*Nocardioides* sp.	34.640 ± 1.110^c^	23.095 ± 1.541 b-d	1.195 ± 0.121 b-d	1.068 ± 0.109^ab^	52.40 ± 1.19^d^	67.96 ± 4.11^c^
5		31.770 ± 1.200^d^	25.215 ± 1.849 a-c	1.245 ± 0.052 a-c	1.045 ± 0.097^bc^	52.92 ± 0.98^d^	59.69 ± 2.40^d^
10		36.723 ± 0.746^bc^	25.728 ± 2.576^ab^	1.300 ± 0.086^ab^	1.073 ± 0.121^ab^	64.49 ± 2.39^b^	60.56 ± 2.71^d^
0	BCFE	38.163 ± 0.896^b^	26.115 ± 2.357^ab^	1.318 ± 0.087^ab^	1.203 ± 0.068^ab^	56.06 ± 2.97^c^	71.91 ± 2.23^b^
5		42.003 ± 0.978^a^	28.953 ± 3.570^a^	1.348 ± 0.057^a^	1.218 ± 0.093^a^	58.75 ± 2.98^c^	65.56 ± 0.79^c^
10		42.013 ± 0.834^a^	28.758 ± 2.511^a^	1.315 ± 0.097^ab^	1.208 ± 0.059^ab^	73.58 ± 0.80^a^	77.17 ± 1.01^a^
0	Control	26.380 ± 1.586^e^	21.308 ± 2.497^de^	1.025 ± 0.111^e^	0.908 ± 0.162^c^	45.00 ± 2.21^e^	50.59 ± 2.72^f^
5		24.630 ± 2.994^ef^	18.370 ± 1.823^e^	1.108 ± 0.079^de^	0.890 ± 0.109^c^	51.87 ± 2.73^d^	55.88 ± 2.63^e^
10		23.123 ± 2.486^f^	21.823 ± 1.805 c-e	1.135 ± 0.037 c-e	0.893 ± 0.078^c^	51.93 ± 1.70^d^	57.86 ± 1.93^de^
	Mean square error	2.593	5.539	0.007	0.011	4.644	6.093
	*R*^2^	0.960	0.0727	0.673	0.670	0.947	0.932

*Values in the same data series represented by different letters are significantly different (*p* = 0.05) in Duncan’s multiple-range test. ^*a*^Concentration in milligram per gram of fresh tissue.*

#### Total Protein Content

Under salinity stress of 5 and 10 dS m^–1^, BCFE priming enhanced protein content up to 42 mg g^–1^ in fresh shoot and 28 mg g^–1^ in fresh root tissue, respectively. Bacterial inoculation resulted in 31.77 mg protein g^–1^ at 5 dS m^–1^ and 36.72 mg g^–1^ at 10 dS m^–1^ fresh shoot tissue. Saline condition alone significantly lowered the shoot protein content up to 24.63 and 23.12 mg g^–1^ of fresh tissue at 5 and 10 dS m^–1^, respectively, compared to non-saline condition (26.38 mg protein g^–1^ fresh tissue) (Table 3). Protein content from root also varied, and seed priming with BCFE performed better than the control. Both microbial inoculation and BCFE priming improved protein content in the shoot and root tissues when the seedlings were under saline condition.

#### Accumulation of Soluble Sugars

The increase in sugar content in plant parts at high salinity stress indicated altered osmotic changes as a mechanism toward stress adaptation. Maximum sugar content was found in BCFE-primed seedlings (73.58 and 77.17 mg g^–1^ fresh tissue, respectively) at 10 dS m^–1^. Compared to BCFE-primed seedlings, the bacteria-inoculated seedlings showed less sugar content, which was much lower in non-inoculated and non-primed seedlings (control) (Table 3).

#### Total Phenolic Content

In BCFE-primed seedlings, shoot and root accumulated a high content of total phenolics (1.348 and 1.218 mg g^–1^ fresh tissue, respectively) (Table 3). The bacteria-inoculated seedlings showed TPC of 1.245 and 1.3 mg g^–1^ of fresh shoot tissue and 1.045 and 1.073 mg g^–1^ of fresh root tissue, respectively, at 5 and 10 dS m^–1^. It remained minimum (1.108 and 1.135 mg g^–1^) in fresh shoot tissue and (0.890 and 0.893 mg g^–1^) in fresh root tissue, respectively, at 5 and 10 dS m^–1^ in non-inoculated and non-primed seedlings.

#### Antioxidant Enzyme Activity in Wheat Seedlings

Bacterial inoculation and BCFE priming actively enhanced the level of antioxidative plant enzymes, namely, CAT, SOD, POD, and APX (Table 4). Salinity stress (5 and 10 dS m^–1^) maximally enhanced the CAT activity in shoot tissues of the plants primed with BCFE (0.039 μM of H_2_O_2_ reduced mg^–1^ protein min^–1^). Bacterial inoculation, however, showed a low CAT activity in the shoot. However, in the root, a reverse trend of CAT activity was observed. Both the microbe-inoculated and the BCFE-primed seedlings showed a high activity than the non-inoculated control plants. SOD was also enhanced due to bacterial inoculation and BCFE priming under saline condition (Table 4). In the shoot and the root tissues of BCFE-primed plants, maximum SOD activity was observed (25.48 and 17.09 U mg^–1^ protein at 10 dS m^–1^).

**TABLE 4:** Antioxidant enzyme activity in wheat seedlings treated with *Nocardioides* sp. and bacterial culture filtrate extract (BCFE) under varying salinity stress conditions.

		Catalase^a^		Superoxide dismutase^b^		Peroxidase^c^		Ascorbate peroxidase^d^	
EC (dS m^–1^)	Treatment	Shoot	Root	Shoot	Root	Shoot	Root	Shoot	Root
0	*Nocardioides* sp.	0.022 ± 0.001^e^	0.020 ± 0.001^b^	15.80 ± 1.16^e^	5.51 ± 0.63^e^	0.038 ± 0.001^bc^	0.056 ± 0.002^b^	0.122 ± 0.007^bc^	0.126 ± 0.006^cd^
5		0.030 ± 0.001^c^	0.013 ± 0.001^e^	20.74 ± 0.98^c^	8.56 ± 0.88^cd^	0.035 ± 0.001^cd^	0.052 ± 0.002^c^	0.127 ± 0.005^b^	0.139 ± 0.011^c^
10		0.024 ± 0.001^d^	0.017 ± 0.001^c^	18.89 ± 0.94^d^	13.62 ± 0.49^b^	0.039 ± 0.001^bc^	0.047 ± 0.002^d^	0.125 ± 0.008^bc^	0.115 ± 0.010^d^
0	BCFE	0.027 ± 0.001^b^	0.032 ± 0.001^a^	22.64 ± 1.35^b^	9.06 ± 1.48^cd^	0.041 ± 0.005^ab^	0.071 ± 0.002^a^	0.134 ± 0.016^b^	0.180 ± 0.012^a^
5		0.039 ± 0.001^a^	0.018 ± 0.001^c^	23.46 ± 0.60^b^	9.78 ± 1.16^c^	0.044 ± 0.004^a^	0.072 ± 0.002^a^	0.187 ± 0.008^a^	0.135 ± 0.007^c^
10		0.039 ± 0.001^a^	0.015 ± 0.001^d^	25.48 ± 0.63^a^	17.09 ± 0.85^a^	0.043 ± 0.002^a^	0.054 ± 0.002^bc^	0.136 ± 0.007^b^	0.159 ± 0.008^b^
0	Control	0.019 ± 0.001^f^	0.013 ± 0.001^de^	11.57 ± 0.91^f^	3.47 ± 0.74^f^	0.032 ± 0.003^d^	0.038 ± 0.002^e^	0.101 ± 0.006^d^	0.100 ± 0.007^e^
5		0.020 ± 0.002^f^	0.012 ± 0.001^e^	14.95 ± 1.67^e^	7.47 ± 2.04^d^	0.032 ± 0.003^d^	0.044 ± 0.005^d^	0.112 ± 0.011^cd^	0.077 ± 0.005^f^
10		0.020 ± 0.001^ef^	0.013 ± 0.001^e^	15.46 ± 2.25^e^	8.47 ± 1.01^cd^	0.032 ± 0.003^d^	0.016 ± 0.002^f^	0.122 ± 0.006^bc^	0.093 ± 0.007^e^
Mean square error		1.30E-006	1.14E-006	1.610	1.277	7.23E-006	4.09E-00	7.97E-005	7.25E-005
*R*^2^		0.983	0.976	0.941	0.939	0.794	0.998	0.895	0.946

*Values in the same data series represented by different letters are significantly different (*p* = 0.05) in Duncan’s multiple-range test. ^*a*^Millimolar of H_2_O_2_ reduced per milligram of protein per minute. ^*b*^Units of enzyme per milligram of protein. ^*c*^Specific enzyme activity per milligram of protein. ^*d*^Millimolar of ascorbate reduced per milligram of protein per minute.*

Moderate saline condition (5 dS m^–1^) increased the level of POD in shoot and root tissues primed with BCFE (0.044 and 0.072 specific enzyme activity mg^–1^ protein, respectively). Bacterial inoculation also enhanced the POD level in plants grown under salinity condition. Priming enhanced the APX activity in shoot at 5 dS m^–1^ (Table 4). Under salinity condition, the activity was low in the root tissues of the primed seedlings. We observed that priming significantly enhanced the activity of CAT, SOD, POD, and APX enzymes, which was low in bacteria-inoculated plants and the control.

#### Gene Expression

**FIGURE 3:**
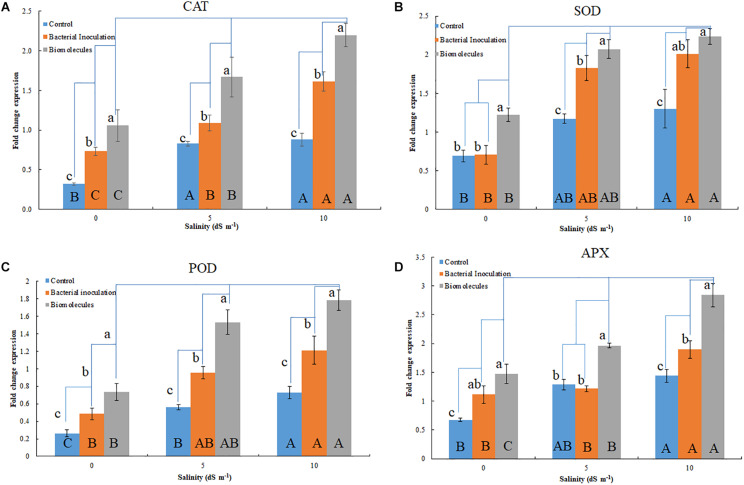
Quantitative gene expression of antioxidant enzymes; *CAT* **(A)**, *MnSOD* **(B)**, *POD* **(C)**, and *APX* **(D)** in plants inoculated with *Nocardioides* strain and bacterial culture filtrate extract. Lowercase indicate comparison within a group (salinity level); while, the uppercase indicates comparison between groups (salinity levels).

The antioxidant genes were upregulated in the seedlings having salinity stress (Figures 3A–D). Although bacterial inoculation significantly enhanced gene expression over the control, the extent of upregulation was quite less than that induced by BCFE priming. The expression of *CAT* gene increased with the increasing stress condition in both the bacterial inoculation and BCFE priming at all levels of stress. However, a plateau was observed at 5 dS m^–1^, indicating no further upregulation of gene expression (Figure 3A). The expression of *MnSOD* and *POD* genes exhibited a marked upregulation at 5 dS m^–1^ that continued at 10 dS m^–1^ (Figures 3A,B). *APX* exhibited a characteristic trend in the control, and in the treatments the gene was intensively upregulated at 5 dS m^–1^ (Figure 3D).

#### Correlation Analysis

**FIGURE 4:**
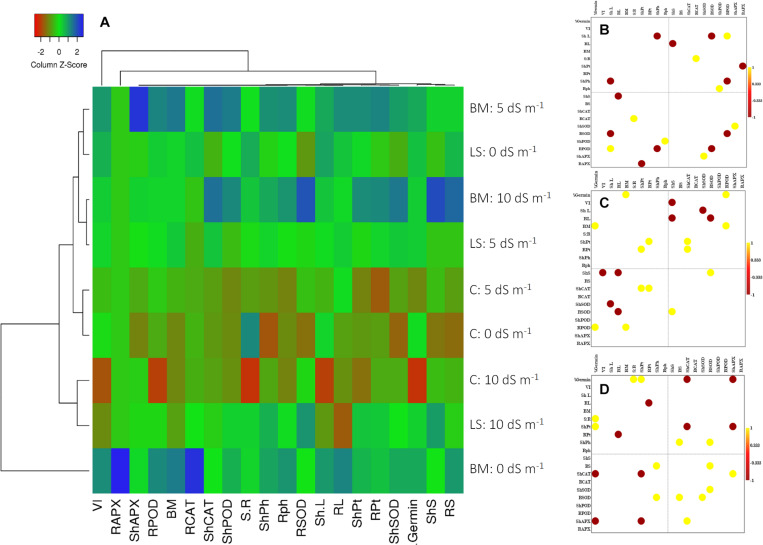
Clustered heat map **(A)** and Pearson’s correlation plots of the measured physicochemical traits of wheat seedlings under the influence of treatments with live strain **(B)** and bacterial culture filtrate extract **(C)** and control **(D)**. Values with *p* ≤ 0.05 are included in the correlation plots.

The cluster heat map prominently reflected physiological and biochemical responses of wheat seedlings inoculated with *Nocardioides* sp. NIMMe6, primed with BCFE and grown under saline condition (Figure 4A). Under non-saline condition (control), the performance of BCFE priming was better than the bacterial inoculation. Low scores (tending toward red) for several studied parameters of non-inoculated and non-primed plants indicated a higher performance of BCFE priming and bacterial inoculation on wheat. Pearson’s correlation analysis revealed a complex intertwined physicochemical trait (Figures 4B–D and Supplementary Tables S1A–C). Phenotypic traits showed a positive correlation of root length with vigor index (*r* = 0.974 and 0.921, respectively) due to the influence of bacterial inoculation, which further enhanced the correlation between biomass and VI (*r* = 0.916). A positive correlation between sugar content in root with germination and POD activity in root with germination, vigor, and shoot and root length was seen due to the bacterial inoculation (Figure 4B and Supplementary Table S1A). Total phenolic content in shoot exhibited a strong negative correlation with shoot length (*r* = −0.998; Figure 4B). CAT activity in root, POD in shoot and root, and APX in shoot showed a significant (*p* ≤ 0.05) positive correlation with shoot–root ratio, total phenolic content in root, shoot length, and SOD activity in shoot, respectively. Such positive correlations reflected a synergistic role of antioxidant enzymes and total phenolics in imparting important plant functions to alleviate abiotic stress. In BCFE-primed plants, biomass and germination (%), protein content in root and shoot, and CAT activity were positively correlated. SOD activity in root was positively linked with sugar content in shoot, while that of POD in root had a positive correlation with germination and biomass, respectively (Figure 4C and Supplementary Table S1B). Such correlations reflected an interlinked associative role of antioxidant and physicochemical status in the shoot and root development in bacteria-inoculated and/or BCFE-primed plants grown under saline conditions.

## Discussion

Methylotrophic bacteria are important members of the phyllosphere community (Iguchi et al., 2015). They particularly inhabit plant leaf surface that facilitates the availability of methanol by virtue of stomatal leakage (Fall and Benson, 1996; Abanda-Nkpwatt et al., 2006). Previously, we have reported pink-pigmented methylotrophic bacteria from the phyllosphere of different crop plants such as sugarcane, pigeon pea, mustard, potato, and radish (Meena et al., 2012). In the present work, we isolated and characterized methylotrophic bacteria from the leaf surface of soybean, a crop of high commercial importance in India. The work was in continuation of our earlier efforts to obtain beneficial methylotrophic plant-growth-promoting rhizobacterial (PGPR) organisms from the phyllosphere of crop plants. The common association of colonizing bacterial species on the plant phyllosphere (Sy et al., 2005) and the potential role of methylotrophic species as PGPRs and mitigators of abiotic stresses in crop plants have been described (Kumar et al., 2019).

The PGP capabilities of methylotrophic bacteria are mainly attributed to their unique ability to synthesize a diverse array of biomolecules (Delmotte et al., 2009). These organisms secrete signaling molecules, phytohormones, vitamin B12, polysaccharides, and osmoprotectants into their habitats (Omer et al., 2004; Bustillos-Cristales et al., 2017). Plants in association with these microorganisms can get strength to sustain under biotic and abiotic stress conditions due to the impact of the phytohormones (Ojuederie et al., 2019). However, investigations that provide direct evidence on the beneficial impact of biomolecule-rich BCFE from methylotrophic bacteria on growth promotion and stress mitigation in crop plants are lacking.

We identified ***Nocardioides*** sp. NIMMe6 on the basis of 16S rRNA sequence similarity. The multiple carbon source utilization profile of this bacterium indicated its metabolic plasticity for better survival under nutritionally diverse habitats (Bochner, 2009). The capability of the isolate to actively utilize hexose sugars, amino acids, carboxylic acid ester, and fatty acids indicated that the bacterium can successfully inhabit chemically diverse habitats and thus can flourish in the dynamic environment of plant rhizosphere where a rich pool of metabolites exists (Berendsen et al., 2012). The actinobacterium ***Nocardioides*** sp. NIMMe6 showed specific PGP traits like siderophore and phytohormone production and survival under N-deficient condition which encouraged its impact assessment on wheat plants. A probable mechanism of SA and IAA production is depicted in Figure 2. The role of IAA in seed germination and development under salinity stress is known (Sorty et al., 2016; Korver et al., 2018). SA is a phenolic acid widely produced in both prokaryotes and plants. SA activity in plants is mainly linked as a messenger for triggering local, and systemic acquired resistance under stress conditions (Conrath, 2006; An and Mou, 2011). We identified both IAA and SA in the culture filtrate of ***No****c****a****r****dioides*** sp. using HPLC and MS (Supplementary Figure S1). This unique metabolic feature of the bacterium further endorsed its selection as a candidate strain for BCFE production in this study. The salt tolerance ability of the bacterium was the additional beneficial trait for applying it on wheat, a crop reported to suffer from saline conditions and whose early developmental stages, including the germination stage, are more vulnerable (Oyiga et al., 2016). Thus, there were reasonable reasons to assess the impact of inoculation of the bacterium and its culture filtrate extract on the wheat plants grown under saline conditions. Several workers have reported the impact of microbial inoculants on crop plants grown in soils of various EC levels. Agronomically, a soil having an EC value exceeding 4 dS m**^–^**^1^ of its saturated extract at room temperature and having exchangeable sodium of 15% is supposed to be saline (Yamaguchi and Blumwald, 2005). At this EC value of the soil, most crops show negative growth performance, although even lower ECs also exhibit yield reduction in crops (Manchanda and Garg, 2008). The positive impact of the inoculation of ***Glomus intraradices*** on bean (***Phaseolus vulgaris***) grown in soil of EC value 3.1 dS m**^–^**^1^, ***Bacillus megaterium*** on maize grown in soil of EC 2.59 dS m**^–^**^1^, and arbuscular mycorrhiza on ***Jatropha curc****a****s*** grown in soils ranging from 1.7 to 8.5 dS m^–1^. EC has been described (Dodd and Pérez-Alfocea, 2012). The comparative growth and development profile of wheat genotypes at different EC levels ranging from 0 to 12 dS m**^–^**^1^ was also evaluated by Kumar et al. (2017). Loss in germination percentage to the tune of 35–44% was recorded at 9 and 12 dS m**^–^**^1^ EC, respectively, in wheat varieties. As per Food and Agriculture Organization reports, the tolerance level of wheat is reported to be^2^ 6 dS m**^–^**^1^. Therefore, the assessment of the impact of microbial inoculation and BCFE in wheat at moderate, 5 dS m**^–^**^1^, to relatively high EC level of 10 dS m**^–^**^1^ seems agronomically rightful and appropriate.

Inoculation of bacterium and priming of BCFE improved all plant parameters studied for growth and development of seedlings. Seedlings grown under saline conditions responded against stress in terms of low germination, vigor index, shoot and root length, and biomass accumulation. In general, a decrease in phenotypic characters like seedling biomass, moisture content, seed germination, and vigor index following salinity stress has been reported as a natural phenomenon (Hasanuzzaman et al., 2013). The impact of bacterial inoculation and BCFE priming significantly reversed the declining growth parameters among seedlings grown under stress conditions (5 and 10 dS m^–1^). With increasing salinity condition, the BCFE-primed wheat seedlings exhibited better physiological performance (germination, vigor index, shoot and root length, and biomass), indicating a significant role of BCFE priming in strengthening the seedlings against salinity stress. The impact is supposed to be due to the direct implications of the metabolites reported in the BCFE.

Cell-free inoculants have been considered as upcoming tools to mitigate abiotic stress(es) under a changing climate scenario (Bashan et al., 2016; Vassilev et al., 2017). Due to their characteristic ability to produce a variety of biomolecules, including plant growth hormones, siderophores, vitamins, phenolic compounds, volatiles, a variety of enzymes, and biocontrol agents, PGP microbes can be potential candidates toward designing cell-free inoculae (Sorty et al., 2016; Sreevidya et al., 2016; Mendes et al., 2017). The bacterium *Nocardioides* sp. NIMMe6 secreted into the culture filtrate IAA and SA. Both of these metabolites have a significant role in plant growth promotion (Egamberdieva et al., 2017) and induction of plant responses against stresses (Ku et al., 2018).

Our results witnessed both evidences. The BCFE which was shown to contain IAA and SA, when used as a priming agent on seeds, resulted in healthy seedlings under saline conditions, and plant growth was even better than bacterial inoculation itself. At the same time, quantitative changes at the level of antioxidant enzyme activity and regulation of antioxidant genes were also observed in BCFE-primed seedlings. It was therefore assumed that BCFE priming not only supported seedling growth but also helped in inducing stress-mitigating mechanisms in plant seedlings. The higher impact of BCFE on root and shoot length, germination, and vigor index in wheat can be attributed to the availability of IAA and SA compounds in higher quantity that favor plant growth. SA is structural component of siderophores (Djavaheri et al., 2012) and helps plants to mount vital responses against abiotic stress conditions (Rivas-San Vicente and Plasencia, 2011). IAA is a plant growth promoter under normal or abiotic stress and helps to maintain hormonal balance for adequate homeostasis (Fu and Harberd, 2003). Inoculation with live bacterial cells showed improved impact as compared to non-inoculated and non-primed plants. The performance of BCFE priming on seeds was comparatively more impactful. This effect may be attributed to the fact that the growth of bacterial inoculant cells under salinity-stressed habitat becomes challenging and the performance of the live inoculant is hampered due to non-favoring cellular development (O’Callaghan, 2016).

Reactive oxygen species that generate oxidative stress in plants (Foyer and Noctor, 2005) critically damage vital organelles and induce cellular apoptosis (Kumar et al., 2015). The overall oxidative challenge is maintained in plants through a delicate balance between ROS formation and concurrent scavenging by enzymatic and non-enzymatic antioxidants (Keunen et al., 2013). The production and the accumulation of phenolic compounds and antioxidant enzymes and the activation of the glutathione system that actively scavenge ROS to prevent consequent damage to cellular components are some of the known mechanisms to overcome stresses (Blokhina et al., 2003). Although plants respond to abiotic stresses by inducing various intrinsic mechanisms within their cells, microbial inoculation leads to enhanced mechanisms that significantly overcome ROS accumulation (Meena et al., 2017). The antioxidant enzymes in plants challenged with abiotic stress reflect cumulative enzymatic response in terms of cellular oxidative stress management. To measure superoxide radical management in tissues, we monitored the activity of SOD enzyme. Similarly, the activity of ascorbate peroxidase, peroxidase, and catalase reflected the efficiency of peroxide (H_2_O_2_) management in wheat seedlings under saline conditions. The improved activity of SOD, CAT, POD, and APX in both root and shoot tissues reflected activated enzymatic mechanisms that help plants to reduce the severity of salinity stress. The increased level of SOD indicated an antioxidant enzyme-based management of superoxide radicals. Similarly, treatment-dependent alterations in the expression of CAT in plants grown under stress were observed (Scandalios, 2005). Our report on the enhanced level of antioxidant enzyme activity under salinity condition aligns with the previous observations of Yang et al. (2008). BCFE priming significantly induced CAT in shoot at a higher salinity level. The enhanced activity of APX and POD also helped in antioxidant enzyme-based management of oxidative damage in wheat.

The management of a plant’s stress level is also achieved by the regulation of various genes that code for the antioxidant enzymes CAT, SOD, APX, and POD to detoxify oxidative radicals (Xie et al., 2019). The upregulation of gene transcripts related to the antioxidant enzymes in inoculated and primed wheat was observed. The result of the upregulation of the four genes certifies the beneficial role of *Nocardioides* sp. NIMMe6 inoculation and BCFE priming in wheat plants grown under saline conditions. Induction of plant abiotic stress tolerance due to the overexpression of genes linked with the antioxidant enzyme by microbial inoculation has been reported (Gururani et al., 2013). We have shown that, along with bacterial inoculation on seeds as inoculants, the BCFE from the bacterium can also be used for stress alleviation in wheat seedlings due to its impact on the modulated biochemical and molecular mechanisms in plants. The BCFE showed a relatively better performance over bacterial live cell inoculation under salinity stress.

Potential benefits of microbial inoculation to plants under abiotic stress conditions have been reported (Singh et al., 2011; Nadeem et al., 2014). The limitations of the live cells of microbial inoculants applied under stress conditions that lower down the inoculant performance have also been discussed (Naylor and Coleman-Derr, 2018). The application of BCFE from the potential microbial species, therefore, could be a viable option. From the potential species having the capacity of becoming a microbial inoculant, specific functional secondary metabolite could be produced through induction of metabolic flux under modified culture conditions (Baral et al., 2018). This approach may offer a more practical, efficient, and sustainable alternative to maintain a plant’s performance under unfavorable conditions. However, a critical evaluation under field conditions is necessary to warrant more applicability for recommendation at a large scale.

## Conclusion

We have explored an actinobacterium, *Nocardioides* sp. (LC140963), for *in vitro* production and secretion into the medium of bioactive compounds like salicylic acid and indole acetic acid. These biomolecules were extracted from bacterial culture filtrate and utilized in the form of BCFE for salinity stress mitigation in wheat through seed priming. The findings suggested that the incorporation of BCFE is efficient over microbial inoculation for mitigation of salinity stress in wheat seedlings. Seed priming with BCFE actively enhanced both the physicochemical status and the oxidative enzymes and helped in the modulation of gene in wheat seedlings. Therefore, the results strongly endorse the strategic utilization of BCFE rich in biomolecules secreted by plant-growth-promoting microbial strain(s) for the strategic management of salinity stress tolerance in crop plants. More specific work will further warrant large-scale applicability under field conditions.

## Data Availability Statement

The datasets generated for this study can be found in the DNA data Bank of Japan; LC140963; https://www.ncbi.nlm.nih.gov/nuccore/LC140963.

## Author Contributions

KM conceptualized the work. KM, UB, and AS designed and conducted the experiments. KM, UB, AS, DS, SK, and VG gene expression studies, and biochemical experiments. KM, AS, UB, and GW prepared the draft of the manuscript. All authors edited and improved the manuscript document.

## Conflict of Interest

The authors declare that the research was conducted in the absence of any commercial or financial relationships that could be construed as a potential conflict of interest.

## Footnotes

1 http://www.blast.ncbi.nlm.nih.gov/Blast.cgi

2 http://www.fao.org/3/y4263e/y4263e0e.htm; visited on 20.4.2020

## Supplementary Material

FIGURE S1 Indole-3-acetic acid and salicylic acid were identified from the pool of bacterial culture filtrate extract (BCFE) secreted by the strain using high-performance liquid chromatography and liquid chromatography–mass spectrometry approaches **(A,B)**. The photographs present the morphological appearance of the strain and BCFE diluted in absolute methanol.

FIGURE S2 Growth characteristics of *Nocardioides* sp. under increasing salt stress conditions from 0 to 10% of NaCl.

FIGURE S3 Metabolic behavior of *Nocardioides* sp. in the presence of a variety of carbon sources and inhibitory environments as evident from the Biolog Gen III assay.
