# Supplementary material for: Mitigation of Salinity Stress in Wheat Seedlings Due to the Application of Phytohormone-Rich Culture Filtrate Extract of Methylotrophic Actinobacterium Nocardioides sp. NIMMe6
Source: Front Microbiol. 2020 Sep 18;11:2091. doi: 10.3389/fmicb.2020.02091 (PMC7531191; doi:10.3389/fmicb.2020.02091)
Supplement: Supplementary file 1 [file Table_1.DOCX]

|  | %Germin | VI | Sh L | RL | BM | S:R | ShPt | RPt | ShPh | Rph | ShS | RS | ShCAT | RCAT | ShSOD | RSOD | ShPOD | RPOD | ShAPX | RAPX |
| --- | --- | --- | --- | --- | --- | --- | --- | --- | --- | --- | --- | --- | --- | --- | --- | --- | --- | --- | --- | --- |
| %Germin |  |  |  |  |  |  |  |  |  |  |  |  |  |  |  |  |  |  |  |  |
| VI | **0.902** |  |  |  |  |  |  |  |  |  |  |  |  |  |  |  |  |  |  |  |
| Sh L | **0.963** | **0.985** |  |  |  |  |  |  |  |  |  |  |  |  |  |  |  |  |  |  |
| RL | 0.782 | **0.974** | **0.921** |  |  |  |  |  |  |  |  |  |  |  |  |  |  |  |  |  |
| BM | 0.653 | **0.916** | 0.833 | **0.983** |  |  |  |  |  |  |  |  |  |  |  |  |  |  |  |  |
| S:R | 0.674 | 0.288 | 0.449 | 0.065 | -0.120 |  |  |  |  |  |  |  |  |  |  |  |  |  |  |  |
| ShPt | -0.240 | -0.636 | -0.493 | -0.793 | -0.892 | 0.556 |  |  |  |  |  |  |  |  |  |  |  |  |  |  |
| RPt | -0.989 | -0.828 | -0.912 | -0.681 | -0.533 | -0.776 | 0.093 |  |  |  |  |  |  |  |  |  |  |  |  |  |
| ShPh | -0.976 | -0.974 | -0.998 | -0.898 | -0.801 | -0.498 | 0.444 | **0.934** |  |  |  |  |  |  |  |  |  |  |  |  |
| Rph | 0.022 | -0.412 | -0.249 | -0.607 | -0.743 | 0.754 | **0.965** | -0.170 | 0.194 |  |  |  |  |  |  |  |  |  |  |  |
| ShS | -0.780 | -0.974 | -0.920 | -1.000 | -0.983 | -0.063 | 0.794 | 0.679 | 0.897 | 0.608 |  |  |  |  |  |  |  |  |  |  |
| RS | **0.909** | 0.640 | 0.763 | 0.451 | 0.278 | **0.920** | 0.186 | -0.961 | -0.798 | 0.436 | -0.449 |  |  |  |  |  |  |  |  |  |
| ShCAT | -0.485 | -0.059 | -0.230 | 0.167 | 0.346 | -0.973 | -0.733 | 0.609 | 0.285 | -0.885 | -0.169 | -0.805 |  |  |  |  |  |  |  |  |
| RCAT | 0.630 | 0.233 | 0.396 | 0.008 | -0.177 | **0.998** | 0.603 | -0.738 | -0.448 | 0.790 | -0.006 | 0.896 | -0.985 |  |  |  |  |  |  |  |
| ShSOD | -0.756 | -0.399 | -0.551 | -0.183 | 0.002 | -0.993 | -0.454 | 0.845 | 0.597 | -0.671 | 0.181 | -0.960 | **0.939** | -0.985 |  |  |  |  |  |  |
| RSOD | -0.945 | -0.993 | -0.998 | -0.942 | -0.864 | -0.396 | 0.543 | 0.887 | **0.993** | 0.305 | **0.942** | -0.724 | 0.173 | -0.342 | 0.502 |  |  |  |  |  |
| ShPOD | -0.052 | -0.479 | -0.321 | -0.664 | -0.791 | 0.703 | **0.982** | -0.096 | 0.267 | **0.997** | 0.665 | 0.368 | -0.848 | 0.742 | -0.614 | 0.375 |  |  |  |  |
| RPOD | **0.968** | **0.982** | **1.000** | **0.913** | 0.822 | 0.466 | -0.476 | -0.920 | -0.999 | -0.230 | -0.912 | 0.775 | -0.249 | 0.415 | -0.567 | -0.997 | -0.302 |  |  |  |
| ShAPX | -0.737 | -0.373 | -0.527 | -0.154 | 0.031 | -0.996 | -0.480 | 0.829 | 0.574 | -0.692 | 0.152 | -0.951 | **0.948** | -0.989 | **1.000** | 0.476 | -0.636 | -0.543 |  |  |
| RAPX | 0.297 | 0.681 | 0.545 | 0.828 | **0.917** | -0.505 | -0.998 | -0.153 | -0.496 | -0.948 | -0.829 | -0.127 | 0.691 | -0.554 | 0.400 | -0.592 | -0.969 | 0.528 | 0.426 |  |

**Table S1a:** Correlation among measured characteristics: Wheat seed-inoculation with *Nocardioides* sp. live strain.

**Table S1b:** Correlation among measured characteristics: Wheat seed-primed with BCFE.

|  | %Germin | VI | Sh L | RL | BM | S:R | ShPt | RPt | ShPh | Rph | ShS | RS | ShCAT | RCAT | ShSOD | RSOD | ShPOD | RPOD | ShAPX | RAPX |
| --- | --- | --- | --- | --- | --- | --- | --- | --- | --- | --- | --- | --- | --- | --- | --- | --- | --- | --- | --- | --- |
| %Germin |  |  |  |  |  |  |  |  |  |  |  |  |  |  |  |  |  |  |  |  |
| VI | **0.987** |  |  |  |  |  |  |  |  |  |  |  |  |  |  |  |  |  |  |  |
| Sh L | **0.951** | **0.989** |  |  |  |  |  |  |  |  |  |  |  |  |  |  |  |  |  |  |
| RL | **0.996** | **0.997** | **0.973** |  |  |  |  |  |  |  |  |  |  |  |  |  |  |  |  |  |
| BM | **0.998** | **0.976** | **0.932** | **0.990** |  |  |  |  |  |  |  |  |  |  |  |  |  |  |  |  |
| S:R | -0.918 | -0.841 | -0.750 | -0.881 | -0.938 |  |  |  |  |  |  |  |  |  |  |  |  |  |  |  |
| ShPt | -0.502 | -0.636 | -0.745 | -0.573 | -0.453 | 0.117 |  |  |  |  |  |  |  |  |  |  |  |  |  |  |
| RPt | -0.446 | -0.585 | -0.701 | -0.519 | -0.395 | 0.053 | **0.998** |  |  |  |  |  |  |  |  |  |  |  |  |  |
| ShPh | 0.569 | 0.428 | 0.287 | 0.499 | 0.614 | -0.849 | 0.425 | 0.482 |  |  |  |  |  |  |  |  |  |  |  |  |
| Rph | 0.189 | 0.027 | -0.124 | 0.106 | 0.243 | -0.564 | 0.754 | 0.795 | **0.915** |  |  |  |  |  |  |  |  |  |  |  |
| ShS | -0.990 | -1.000 | -0.985 | -0.998 | -0.980 | 0.852 | 0.620 | 0.569 | -0.447 | -0.047 |  |  |  |  |  |  |  |  |  |  |
| RS | -0.838 | -0.738 | -0.627 | -0.789 | -0.867 | **0.986** | -0.052 | -0.115 | -0.926 | -0.695 | 0.751 |  |  |  |  |  |  |  |  |  |
| ShCAT | -0.512 | -0.645 | -0.753 | -0.582 | -0.463 | 0.128 | **1.000** | **0.997** | 0.415 | 0.747 | 0.629 | -0.040 |  |  |  |  |  |  |  |  |
| RCAT | 0.642 | 0.758 | 0.848 | 0.704 | 0.598 | -0.285 | -0.985 | -0.972 | -0.264 | -0.631 | -0.745 | -0.119 | -0.987 |  |  |  |  |  |  |  |
| ShSOD | -0.960 | -0.993 | -1.000 | -0.980 | -0.943 | 0.769 | 0.724 | 0.679 | -0.316 | 0.094 | **0.990** | 0.651 | 0.732 | -0.831 |  |  |  |  |  |  |
| RSOD | -0.997 | -0.997 | -0.973 | -1.000 | -0.991 | 0.882 | 0.570 | 0.517 | -0.501 | -0.109 | **0.998** | 0.791 | 0.580 | -0.702 | **0.979** |  |  |  |  |  |
| ShPOD | -0.345 | -0.493 | -0.619 | -0.422 | -0.292 | -0.057 | **0.985** | **0.994** | 0.575 | 0.857 | 0.475 | -0.224 | **0.983** | -0.941 | 0.594 | 0.420 |  |  |  |  |
| RPOD | **0.999** | **0.978** | **0.934** | **0.991** | **1.000** | -0.936 | -0.459 | -0.401 | 0.609 | 0.237 | -0.982 | -0.864 | -0.469 | 0.603 | -0.945 | -0.991 | -0.298 |  |  |  |
| ShAPX | 0.471 | 0.321 | 0.175 | 0.395 | 0.519 | -0.783 | 0.527 | 0.580 | **0.993** | **0.955** | -0.340 | -0.876 | 0.517 | -0.374 | -0.205 | -0.398 | 0.666 | 0.514 |  |  |
| RAPX | -0.025 | 0.137 | 0.285 | 0.058 | -0.081 | 0.421 | -0.852 | -0.884 | -0.836 | -0.986 | -0.117 | 0.567 | -0.846 | 0.750 | -0.256 | -0.056 | -0.930 | -0.075 | -0.894 |  |

**Table S1c:** Correlation among measured characteristics: Control (Wheat seed- non-inoculated, non-primed).

|  | %Germin | VI | Sh L | RL | BM | S:R | ShPt | RPt | ShPh | Rph | ShS | RS | ShCAT | RCAT | ShSOD | RSOD | ShPOD | RPOD | ShAPX | RAPX |
| --- | --- | --- | --- | --- | --- | --- | --- | --- | --- | --- | --- | --- | --- | --- | --- | --- | --- | --- | --- | --- |
| %Germin |  |  |  |  |  |  |  |  |  |  |  |  |  |  |  |  |  |  |  |  |
| VI | **0.925** |  |  |  |  |  |  |  |  |  |  |  |  |  |  |  |  |  |  |  |
| Sh L | **0.976** | **0.985** |  |  |  |  |  |  |  |  |  |  |  |  |  |  |  |  |  |  |
| RL | 0.126 | 0.494 | 0.338 |  |  |  |  |  |  |  |  |  |  |  |  |  |  |  |  |  |
| BM | -0.074 | 0.311 | 0.144 | **0.980** |  |  |  |  |  |  |  |  |  |  |  |  |  |  |  |  |
| S:R | **0.998** | 0.897 | **0.960** | 0.059 | -0.141 |  |  |  |  |  |  |  |  |  |  |  |  |  |  |  |
| ShPt | **1.000** | **0.936** | **0.983** | 0.157 | -0.043 | **0.995** |  |  |  |  |  |  |  |  |  |  |  |  |  |  |
| RPt | -0.065 | -0.439 | -0.279 | -0.998 | -0.990 | 0.003 | -0.095 |  |  |  |  |  |  |  |  |  |  |  |  |  |
| ShPh | -0.978 | -0.824 | -0.909 | 0.085 | 0.282 | -0.990 | -0.971 | -0.147 |  |  |  |  |  |  |  |  |  |  |  |  |
| Rph | 0.822 | 0.544 | 0.679 | -0.461 | -0.629 | 0.859 | 0.804 | 0.515 | -0.923 |  |  |  |  |  |  |  |  |  |  |  |
| ShS | -0.904 | -0.673 | -0.790 | 0.310 | 0.493 | -0.931 | -0.890 | -0.369 | **0.974** | -0.987 |  |  |  |  |  |  |  |  |  |  |
| RS | -0.983 | -0.840 | -0.921 | 0.056 | 0.254 | -0.993 | -0.977 | -0.118 | **1.000** | -0.912 | **0.967** |  |  |  |  |  |  |  |  |  |
| ShCAT | -0.997 | -0.950 | -0.990 | -0.199 | 0.000 | -0.990 | -0.999 | 0.138 | **0.959** | -0.778 | 0.870 | **0.967** |  |  |  |  |  |  |  |  |
| RCAT | 0.439 | 0.064 | 0.234 | -0.836 | -0.929 | 0.498 | 0.411 | 0.868 | -0.618 | 0.872 | -0.781 | -0.595 | -0.371 |  |  |  |  |  |  |  |
| ShSOD | -0.946 | -0.753 | -0.854 | 0.201 | 0.392 | -0.966 | -0.936 | -0.261 | **0.993** | -0.962 | 0.994 | **0.989** | **0.920** | -0.705 |  |  |  |  |  |  |
| RSOD | -0.967 | -0.796 | -0.888 | 0.132 | 0.327 | -0.982 | -0.958 | -0.194 | **0.999** | -0.941 | **0.983** | **0.997** | **0.945** | -0.655 | **0.998** |  |  |  |  |  |
| ShPOD | -0.329 | -0.663 | -0.525 | -0.978 | -0.918 | -0.264 | -0.358 | **0.964** | 0.122 | 0.268 | -0.107 | 0.151 | 0.397 | 0.705 | 0.006 | 0.075 |  |  |  |  |
| RPOD | 0.687 | **0.912** | 0.828 | 0.808 | 0.674 | 0.636 | 0.709 | -0.770 | -0.519 | 0.151 | -0.310 | -0.543 | -0.739 | -0.352 | -0.415 | -0.477 | -0.912 |  |  |  |
| ShAPX | -0.999 | -0.942 | -0.985 | -0.172 | 0.027 | -0.994 | -1.000 | 0.111 | **0.967** | -0.795 | 0.883 | **0.974** | **1.000** | -0.397 | **0.930** | **0.954** | 0.372 | -0.720 |  |  |
| RAPX | 0.367 | -0.015 | 0.157 | -0.877 | -0.955 | 0.428 | 0.338 | 0.905 | -0.554 | 0.831 | -0.729 | -0.530 | -0.297 | **0.997** | -0.647 | -0.593 | 0.758 | -0.424 | -0.323 |  |

Strong positive correlations are indicated by bold fonts; while strong negative correlations are indicated by underlines.
