# Supplementary figures and images for: Mitigation of Salinity Stress in Wheat Seedlings Due to the Application of Phytohormone-Rich Culture Filtrate Extract of Methylotrophic Actinobacterium Nocardioides sp. NIMMe6
Source: Front Microbiol. 2020 Sep 18;11:2091. doi: 10.3389/fmicb.2020.02091 (PMC7531191; doi:10.3389/fmicb.2020.02091)

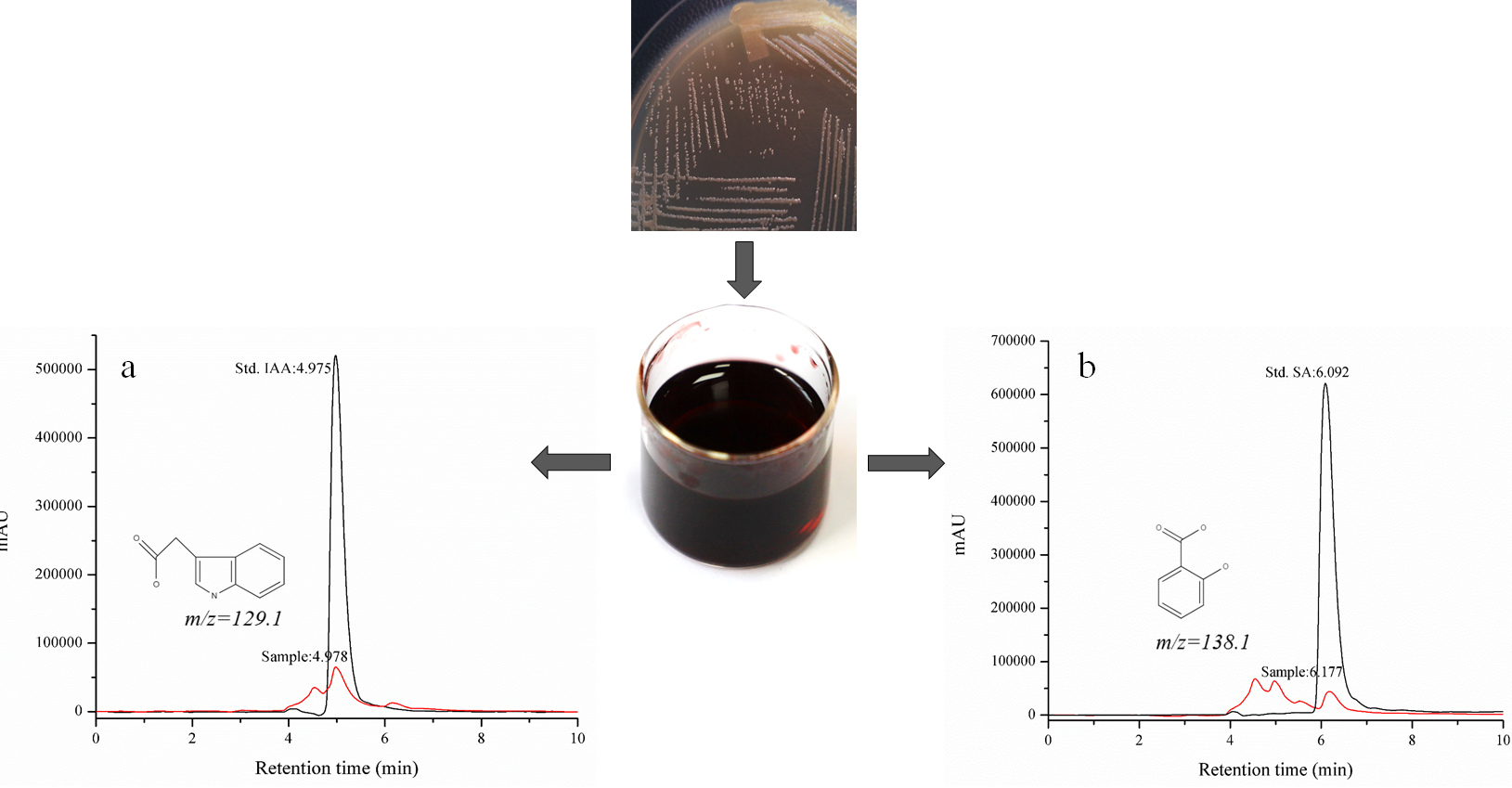

Supplement: FIGURE S1 — Indole-3-acetic acid and salicylic acid were identified from the pool of bacterial culture filtrate extract (BCFE) secreted by the strain using high-performance liquid chromatography and liquid chromatography–mass spectrometry approaches (A,B). The photographs present the morphological appearance of the strain and BCFE diluted in absolute methanol. [file Image_1.TIF]

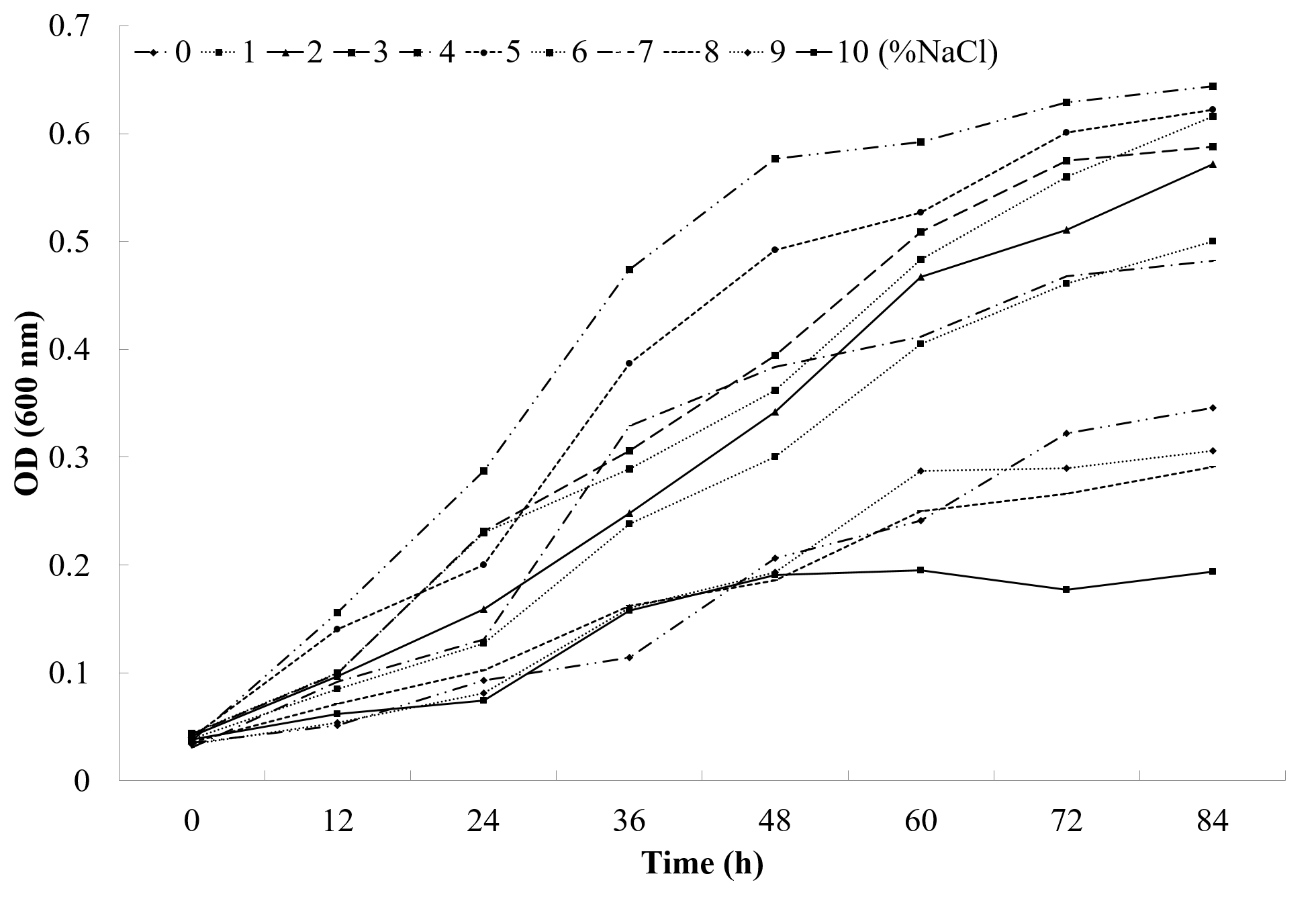

Supplement: FIGURE S2 — Growth characteristics of Nocardioides sp. under increasing salt stress conditions from 0 to 10% of NaCl. [file Image_2.TIF]

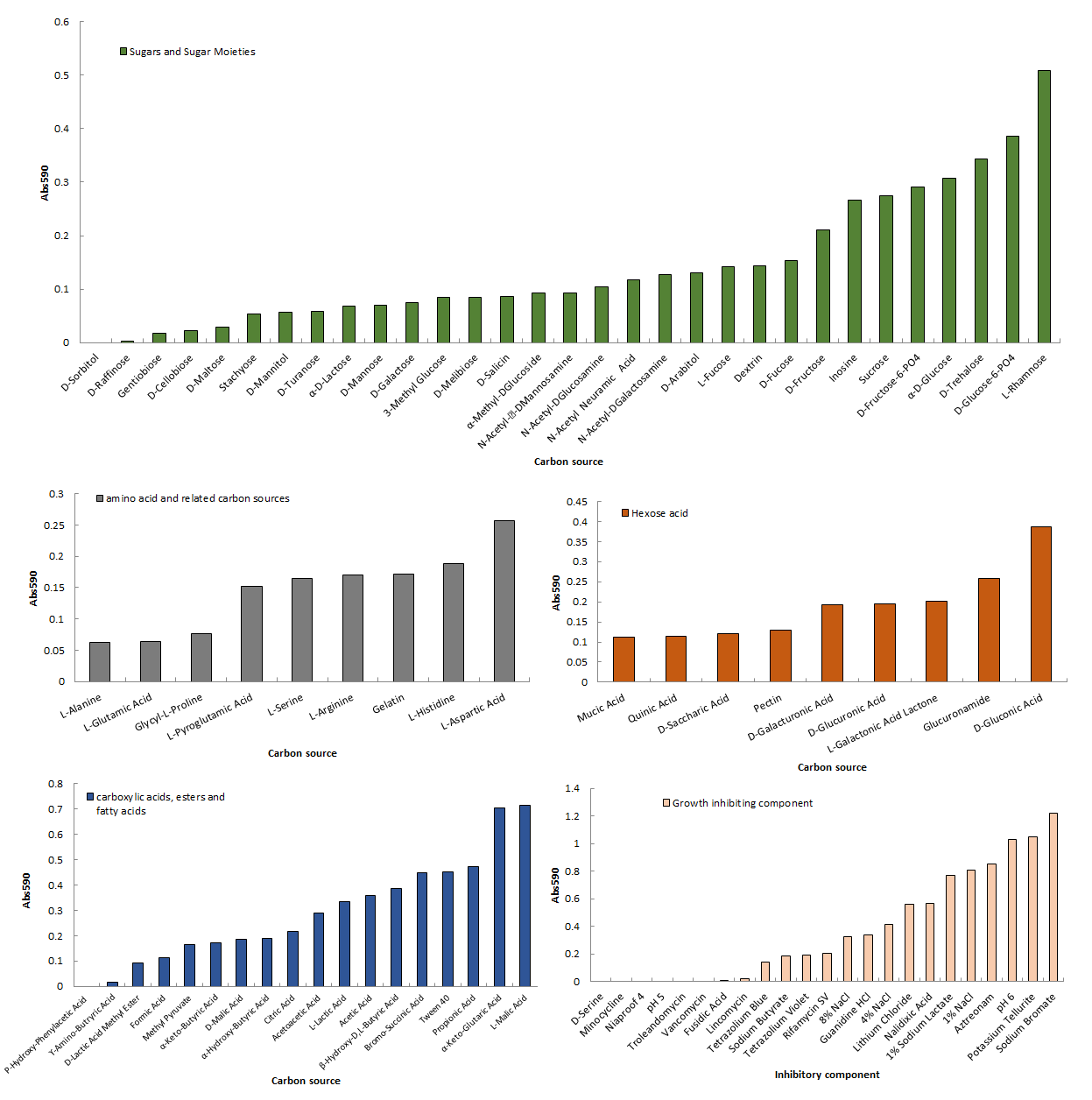

Supplement: FIGURE S3 — Metabolic behavior of Nocardioides sp. in the presence of a variety of carbon sources and inhibitory environments as evident from the Biolog Gen III assay. [file Image_3.TIF]
